# Intra- and interrater reliabilities and a method comparison of 2D and 3D techniques in cadavers to determine sacroiliac screw loosening

**DOI:** 10.1038/s41598-019-40052-4

**Published:** 2019-02-28

**Authors:** Philipp Pieroh, Maximilian Lenk, Tim Hohmann, Ronny Grunert, Daniel Wagner, Christoph Josten, Andreas Höch, Jörg Böhme

**Affiliations:** 1 Department of Orthopaedics, Trauma and Plastic Surgery, University of Leipzig, Liebigstrasse 20, 04103 Leipzig, Germany; 2 Department of Anatomy and Cell Biology, Martin Luther University Halle-Wittenberg, Grosse Steinstrasse 52, 06097 Halle, Saale Germany; 3 Fraunhofer Institute for Machine Tools and Forming Technology IWU, Noethnitzer Strasse 44, 01187 Dresden, Germany; 4 Department of Orthopaedics and Traumatology, University Medical Centre Mainz, Langenbeckstrasse 1, 55131 Mainz, Germany; 5 Present Address: Hospital St. Georg gGmbH, Clinic of Trauma, Orthopaedic and Septic Surgery, Delitzscher Strasse 141, Leipzig, 04129 Germany

## Abstract

Sacroiliac (SI) screw loosening may indicate persistent instability, non-union and contribute to pain. Yet, there is no reliable objective measurement technique to detect and monitor SI screw loosening. In 9 cadaveric pelvises one of two SI screw was turned back approximately 20 mm and subsequently assessed by optical measurement, fluoroscopy and a 3D scan using an image intensifier. CTs were segmented and a contour-based registration of the 3D models and the fluoroscopies was performed to measure SI backing out (X-ray module). Three independent observers performed measurements with three repetitions. Deviation of the measurement techniques to the 3D scan, intra- and interrater reliabilities and method equivalence to the 3D scan were assessed. The X-ray module and two fluoroscopic measurement techniques yielded a difference less than 5 mm compared to the 3D scan and equivalence to the 3D scan. Intrarater reliability was for two observers and almost all techniques very good. Three fluoroscopic measurement techniques and optical measurements displayed a very good interrater reliability. The 3D scan and X-ray module yielded the most precise values for SI screw loosening but only the fluoroscopic measurement of the inlet lateral loosening displayed a good reliability and equivalence to the 3D scan.

## Introduction

Sacroiliac (SI) screw loosening may cause pain and indicate missing fracture healing and persistent instability^1–3^. In the elderly, this is most probably caused by a decreased bone stock in the alar region but also in the sacral body^1,4–7^. The incidence of SI screw loosening with a backing out of the SI screw was described with 2–20% and fractures of the vertical shear type were identified as risk factor^1–3,7–9^. Nonetheless, SI screw loosening did not generally lead to pain or indicate instability^1,3,8^. Especially if the SI screw loosening persists without progress, revision surgery is not required^1,3,8^. Though previous studies described SI screw loosening qualitatively^1–3,5,7^, quantitative measurement techniques were not mentioned bearing the risk to overlook SI screw loosening progression and leading to missing data regarding values indicating clinical relevant SI screw loosening.

The assessment of the healing progress is not only based on the functional outcome, but also radiographic follow-up is of high interest^10^. However, the reliability of radiological measurement methods has to be assessed before correlating functional and radiological outcome^11^.

Radiographic measurement techniques, previously introduced to determine pelvic fracture reduction, revealed a poor level of evidence, missing standardization, reliability assessment and a lack of instructions how to perform the measurement^10^. Lefaivre *et al*.^11^ examined the interrater reliability of some promising radiographic techniques and observed poor to moderate results. Only the cross-measurement technique of Keshishyan *et al*.^12^ assessing pelvic instability yielded excellent results^11^. Especially for the posterior pelvic ring a poor reliability of radiographic measurements was observed^13^. In spine surgery, CT was proposed to assess implant loosening^14^. More sophisticated and expensive examinations such as single photon emission computed tomography (SPECT)/computed tomography (CT) are highly sensitive and specific detecting implant loosening^14,15^. However, the tracer uptake might be increased within the first year after surgery leading to false- positive results^14,15^. Taking the time of SI screw loosening into account (6–69 days) false-positive results might be determined^1^. There is lacking evidence describing techniques to assess specifically SI screw loosening with readily available conventional X-ray.

As yet there are no reliable methods to quantify SI screw loosening, we aimed to develop and study different methods using cadavers. We investigated three-dimensional (3D) scan, optical measurements, a novel developed X-ray module and fluoroscopic measurement techniques to reveal an accurate and reliable measurement technique to detect and monitor SI screw loosening.

## Materials and Methods

### Ethical statement

Cadaver pelvises were obtained from body donors who gave their signed consent for the use of their bodies for educational and research purposes in medical school in accordance to the Saxonian Death and Funeral Act of 1994.

### Cadavers

Nine fresh-frozen cadaveric pelvises (mean age: 85.89 ± 4.96 years; 3 female, 6 male; Supplementary Table S1) were analysed. The complete pelvic ring including the fifth lumbar vertebra was obtained. Surrounding soft tissue and femora were removed and the ligamentous structures were preserved. The pelvises were used beforehand in a biomechanical study of Höch *et al*.^16^ to test the stability of augmented vs. non-augmented SI screws. Therefore, both-sided alar fracture of the sacrum was set with an oscillating saw and the symphysis was cut^16^. In each pelvis, one side was fixed with a single non-augmented fenestrated SI screw in S1 and the other with a single bone cement (3 ml polymethylmethacrylat [PMMA]) augmented fenestrated SI screw in S1. For the present study, the non-augmented SI screw was retracted in each pelvis after biomechanical testing (Supplementary Table S1).

### Scenario definition and SI screw loosening

**Figure 1:**
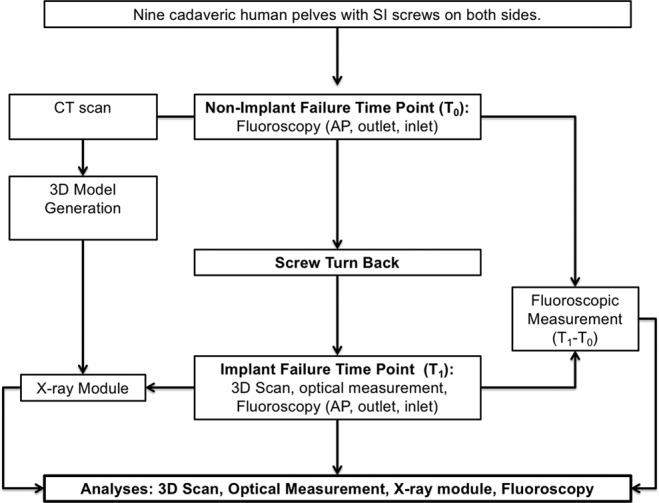
Study protocol and data acquisition. A CT-scan and fluoroscopic images (anterior-posterior [AP], outlet, and inlet view) were obtained at the non-implant failure time point (T_0_) after biomechanical testing^16^. The CT scan was used to generate a 3D model. Subsequently, the non-augmented screw was turned back. Now, at the implant failure time point (T_1_), a 3D scan, an optical measurement, and fluoroscopy were performed. Fluoroscopy was registered to the previously generated 3D model and with the X-ray module the SI screw turn back was computed. The SI screw loosening was calculated (T_1_ − T_0_) using fluoroscopic images and assessed in the 3D scan and the optical measurement.

The position of the non-augmented SI screw was investigated by CT (voxel size: 0.71 × 0.071 × 3 mm, axial orientation, Brilliance, Philips Medical Systems, Cleveland, USA) and fluoroscopic images (anterior-posterior [AP], outlet, and inlet view). This was defined as *non-implant failure* time point (T_0_) (Fig. 1). Then, the non-augmented SI screw was turned back manually approximately 20 mm (18.9 ± 3 mm). This was measured by a parallel-applied Kirschner wire (K-wire) comparable to a measurement with a calliper^13^. Three independent observers at three repetitions performed all below- mentioned measurements.

### 3D Scan based measurements

**Figure 2:**
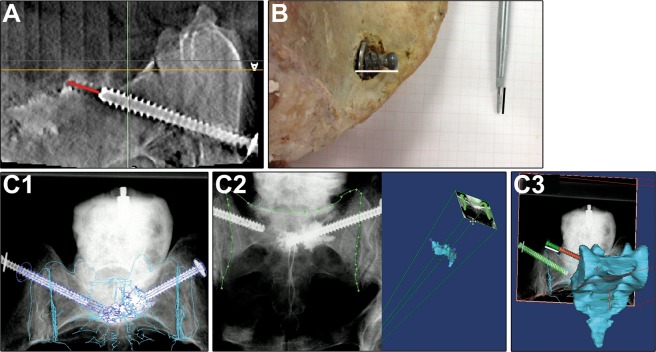
Measurement of SI screw loosening using the 3D scan (**A**), optical measurement (**B**) and the X-ray module (**C**), n = 9. (**A)** SI screw turn back (red line) indicated by the radiolucency was measured in the 3D scan. (**B**) Optical measurement: A K-wire within a drill sleeve was applied parallel to the SI screw. After retraction of the drill sleeve to the screw head, the length of the extruding K-wire was assessed after placing on millimetre paper using ImageJ (black line). (**C**) X ray module: A 3D model based on the CT scan was generated using Mimics and each fluoroscopic image (AP, outlet, inlet) of T_1_ was manually registered to the beforehand-created 3D model (C1). The registration was further enhanced by contour-based registration (C2). One screw model (in red) was adjusted to the screw position of the CT scan, another screw model (green) was positioned according to the fluoroscopic images of T_1_ (green screw). The distance between these screws (white line) was measured.

At T_1_, an axial 3D scan with a voxel size of 0.38 × 0.38 × 1 mm was generated with the image intensifier Ziehm Vision FD Vario 3D^©^ (Ziehm Imaging GmbH, Nürnberg, Germany). The scan centre was set at the anterior-posterior centre of the SI joint of the loosened SI screw. For each pelvis a scan was carried out, exported in DICOM format and imported in Mimics (Version 16, Materialise, Leuven, Belgium). The distance of the SI screw turning back was measured in the slice of the maximum radiolucency (chosen by each observer individually). The turn back was measured from the tip of the screw to the end of the visible radiolucency created by the bone void of the manually turned back SI screw (Fig. 2A, red line)^17^.

### Optical measurement

As mentioned above, the screw turn back was controlled by a parallel set K-wire, in a comparable manner as done with a calliper^13^. Following screw turn back (T_1_), each pelvis was placed on millimetre paper with the SI screw parallel to the millimetre paper. A K-wire in a drill sleeve was set parallel to the turned back SI screw with the tip in contact with the cortical bone. The drill sleeve was retracted up to the level of the screw head, placed on the millimetre paper and photographed (Fig. 2B, black line). SI screw loosening was defined as distance from the tip of the K-wire to the drill sleeve and measured using Image J (ImageJ 1.43, imagej.nih.gov/ij/). Considering the interindividual application of the K-wire and previous obtained similar values for manual and digital measurements^18^, all observers measured the SI screw turn back digitally in photographs.

### 2D/3D Image registration using the X-ray module

DICOM data of the CT scan at T_0_ were imported into Mimics. M.L. segmented manually the cortical bone of the innominate bones, sacrum, fracture fragments and SI screws separately. On the basis of these segmentations the 3D model was generated.

The fluoroscopic images (AP, outlet, inlet) of T_1_ were imported using the X-ray module of Mimics (Fig. 2C). The following steps were performed by each observer for each repetition.

The sacrum, fracture fragments and the non-loosened SI screw were used as reference to match the fluoroscopic images on the 3D model (Fig. 2C1). We used the contour-based registration function of the X-ray module to improve the manual performed overlay of the 3D model and fluoroscopic images (Fig. 2C2). A stereo lithography (STL) file of the SI screw, with the respective length (75–90 mm), was imported and positioned to the SI screw location within the CT scan (T_0_). Subsequently, we duplicated the STL file of the SI screw. The duplicate was then set to the position found in the fluoroscopy (T_1_, Fig. 2C3).

SI screw turn back was determined using the centre of mass translation function of the X-ray module to assess the distance of the two virtual screws.

### Fluoroscopic measurements

To determine SI screw turn back, we used the following workflow after the import and registration of all fluoroscopic images of T_0_ and T_1_:

1. The cranial sacral alae were connected^19^ (Fig. 3 line 1) in AP and outlet views.

**Figure 3:**
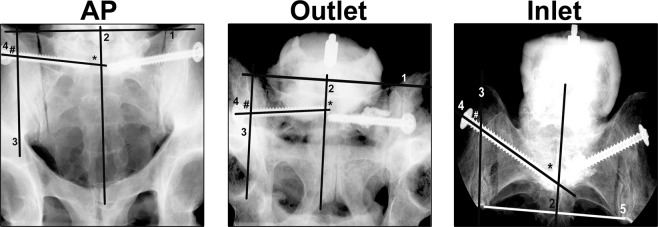
Fluoroscopic Measurements. Line 1 connects the most cranial projections of the sacral alae horizontally (1) in the anterior-posterior (AP) and outlet view. Line 2 runs through the spinous processes of the sacrum, line 3 indicates the lateral borders of the sacral ala and line 4 marks the course of the loosened SI screw. In the inlet view the most ventral parts of the sacral alae were connected horizontally by line 5. Line 2 and 3 were set perpendicular to line 1 in AP and outlet view or respectively to line 5 in the inlet view. Medial loosening (*) was determined on line 4 as distance from the tip of the screw to line 2. The lateral loosening (#) was measured on line 4 as distance from the screw head to line 3.
2. Perpendicular to the cranial connection of the sacral alae, the sacral midline was defined at the level of the sacral processes (Fig. 3, line 2).
3. For the inlet view, the sacral midline (Fig. 3, line 2) was perpendicular to a line connecting the most ventral located portion of the sacral alae (Fig. 3, line 5).
4. Perpendicular to line 1 (AP, outlet) or line 5 (inlet) a line marking the superior lateral border or respectively the lateral border of sacral alae was set (Fig. 3, line 3).
5. The course of the SI screw was marked (Fig. 3, line 4).

Screw turn back was calculated as difference (T_1_ − T_0_) of the values obtained before (T_0_) and after (T_1_) SI screw turn back in each pelvis in AP, inlet and outlet views. The medial loosening (Fig. 3, *) was determined as distance of the SI screw tip to the sacral midline (Fig. 3, line 2). The lateral loosening (Fig. 3, #) was defined as the distance between the screw head and the superior lateral border/lateral border of the sacral alae (Fig. 3, line 3).

Four pelvises were excluded form fluoroscopic measurements because at least one of the used lines was not visualized due to an overlaying polyurethane block fixing the fifth lumbar vertebra or to the limited range of image space. For the remaining imaging methods, all nine pelvises were evaluated.

### Raw data

Descriptive statistics were carried out for all data as mean ± standard deviation (SD) and corresponding range independent of observer and measurement repetition using Graph Pad Prism 7 (GraphPad software, La Jolla, CA, USA). The theoretical accuracy of each method was obtained from the pixel solution of the respective data.

### Differences of the measurement techniques to the 3D scan

Data regardless of observer and repetition were merged and mean values for the 3D scan and other methods were generated for each pelvis specifically. The differences between the 3D scan and each method were calculated for each pelvis as absolute value. The here-obtained data were merged and are presented as mean ± SD including range.

### Intrarater Reliability - ICC (1)

The raw data without generating a mean value were used to study the reliability for each measurement method using a MatLab script (Version R2013a, MathWorks, Natick, Massachusetts, USA).

ICC (1) was chosen in accordance to McGraw and Wong 1996 to estimate the reproducibility of observer data in relation to the performed repetitions^20^. Thus, measurements of each obtained value on the basis of the measurement method from a specific pelvis are interchangeable but the measurements of observers and the other pelvis are not. The grading of Landis and Koch^21,22^ were applied: ICC(1) 1 perfect, 0.81–1 very good, 0.61–0.80 good, 0.41–0.60 moderate, <0.4 poor. The 95% confidence interval (95% CI) was used to define upper (97.5% of values) and lower limit (2.5% of values). If the CI was <0, it was set to 0 related to the definition of the investigated range of ICC (0 to 1). Data are presented as mean and 95% CI.

### Interrater Reliability - ICC (A,1)

Accordingly, to assess intrarater reliability the raw data for each measurement method, pelvis and the three observer data and their repetitions were used. Prior to analyses, a mean value of the three repetitions of each observer for each pelvis and measurement method was generated. The ICC (A,1) was chosen to compare the repeatability between observers^20^. Hence, within the measurement methods the measurements for the pelvis might be changed but the observer data are not interchangeable. The criteria for ICC (A,1) were analysed regarding confidence interval and quality as done for the ICC (1). Data are presented as mean and 95% CI.

### Method Comparison

The 3D scan was defined as “gold standard” as CT is the recommended radiological examination to detect implant related complications in spine surgery^14^.

A mean value of the three repetitions of each observer for each pelvis and measurement method was generated. The generated mean values of each measurement method were subtracted from the mean value of the 3D scan for each pelvis and observer. These difference values were averaged and presented. To verify the application of the 3D scan as “gold standard”, the data of the K-wire measured screw turn back (Supplementary Tables S1 and S4) were included in the method comparison.

A two one-sided test was used estimating equality/divergence of each method to the 3D scan. First, a mean value (1) for all measurement methods was generated for each observer based on the three repetitions for each pelvis. These mean values (1) were pooled for each measurement method and new mean values (2) including standard deviation were generated which were used for the analysis^23^ using a MatLab script. Methods were considered equal if the 95% CI of the measurement method was fully contained in the extreme values (minimum, maximum) of the 3D scan. Values are presented as absolute deviation from “gold standard” (mean and 95% CI). The minimal detectable screw extrusion was determined based on the 95% CI from each method.

## Results

The obtained data of SI screw loosening for each pelvis were merged and the mean ± SD as well as the range are indicated in Supplementary Tables S2 and S3. The theoretical accuracies for all methods were 0.4 mm, calculated on the basis of the respective image solution.

### Differences of the measurement techniques to the 3D scan

The determined SI screw turn back in the 3D scan as well as the differences between the 3D scan and each method are summarized in Table 1.

**Table 1:** Determination of the SI screw loosening in the 3D scan and differences between the 3D scan and each used method given as mean ± SD and range. *n = 9; ^†^n = 5.

Method	Mean ± SD [mm]	Range [mm]
3D Scan*	19.5 ± 2.4	(15.8–23.9)
***Difference to 3D scan***		
Optical Measurement*	6.1 ± 3.1	(1.7–10.5)
X- ray Module*	3.3 ± 1.9	(0.3–6.3)
***Difference 3D scan to fluoroscopic measurements***		
AP medial loosening^†^	7.6 ± 4.4	(2.3–14.5)
AP lateral loosening^†^	5.3 ± 3.6	(0.3–9.0)
Outlet medial loosening^†^	4.8 ± 2.3	(1.5–7.2)
Outlet lateral loosening^†^	9.2 ± 5.6	(0.3–14.2)
Inlet medial loosening^†^	6.9 ± 2.6	(4.5–10.6)
Inlet lateral loosening^†^	4.6 ± 1.8	(2.0–6.6)

None of the used method showed a difference of more than 10 mm compared to the 3D scan. The X-ray module, the medial outlet loosening and the inlet lateral loosening yielded differences less than 5 mm.

### Intrarater Reliability ICC (1)

The ICC (1) using 3D scan was very good for observer 1 and 2 and good for observer 3 (Table 2). The ICC (1) for the optical measurement was very good for all observers. The X-rays module yielded very good ICC (1) for observer 1 and 2 and a moderate ICC (1) for observer 3.

**Table 2:** Intrarater reliability (ICC (1)), mean ICC (1) and 95% CI in parenthesis are given. *n = 9; ^†^n = 5.

Method	Observer 1	Observer 2	Observer 3
	*Mean ICC (1) [95% CI]*	*Mean ICC (1) [95% CI]*	*Mean ICC (1) [95% CI]*
3D Scan*	0.99 [1, 0.96]	0.82 [0.95, 0.56]	0.69 [0.91,0.34]
Optical Measurement*	0.82 [0.95, 0.58]	0.95 [0.99, 0.87]	0.87 [0.96, 0.68]
X- ray Module*	0.91 [0.98, 0.76]	0.93 [0.98, 0.81]	0.46 [0.81, 0.06]
***Fluoroscopic measurements***			
AP medial loosening†	0.91 [0.99, 0.68]	0.90 [0.99, 0.63]	0.69 [0.96, 0.20]
AP lateral loosening†	0.96 [1, 0.83]	0.93 [0.99, 0.74]	0.89 [0.99, 0.60]
Outlet medial loosening†	0.99 [1, 0.994]	0.87 [0.98, 0.55]	0.64 [0.95, 0.13]
Outlet lateral loosening†	0.97 [1, 0.89]	0.96 [0.99, 0.82]	0.96 [1, 0.83]
Inlet medial loosening†	0.68 [0.96, 0.18]	0.88 [0.98, 0.57]	0.29 [0.86, 0]
Inlet lateral loosening†	0.94 [0.99, 0.75]	0.90 [0.99, 0.64]	0.85 [0.98, 0.51]

The following ICC (1) were determined for fluoroscopic measurements: AP medial loosening very good for observer 1 and 2 and good for observer 3, AP lateral loosening very good for all observers, outlet medial loosening was very good for observer 1 and 2 and good for observer 3, outlet lateral loosening very good for all observers, inlet medial loosening for observer 1 good, for observer 2 very good and for observer 3 poor, inlet lateral loosening very good for all observers.

### Interrater Reliability ICC (A,1)

The ICC (A,1) was moderate for the 3D Scan (Table 3), very good for the optical measurement and poor for the X-ray module. Very good ICC (A,1) were obtained for AP lateral, inlet lateral, and outlet lateral loosening using fluoroscopy. The ICC (A,1) was good for AP medial loosening, moderate for outlet lateral loosening, and poor for inlet medial loosening.

**Table 3:** Interrater reliability (ICC (A,1)), mean ICC(A,1) and 95% CI in parenthesis are given. *n = 9; ^†^n = 5.

Method	Mean ICC(A,1) [95% CI]
3D Scan*	0.47 [0.82, 0.09]
Optical Measurement*	0.82 [0.95, 0.49]
X- ray Module*	0.16 [0.66, 0]
***Fluoroscopic measurements***	
AP medial loosening^†^	0.71 [0.96, 0.14]
AP lateral loosening^†^	0.96 [1, 0.80]
Outlet medial loosening^†^	0.43 [0.90, 0]
Outlet lateral loosening^†^	0.97 [1, 0.88]
Inlet medial loosening^†^	0.39 [0.88, 0]
Inlet lateral loosening^†^	0.94 [0.99, 0.78]

### Method Comparison

The mean deviation from the K-wire measured SI screw turn back (Supplementary Table S1) to the 3D scan was 0.6 mm.

**Figure 4:**
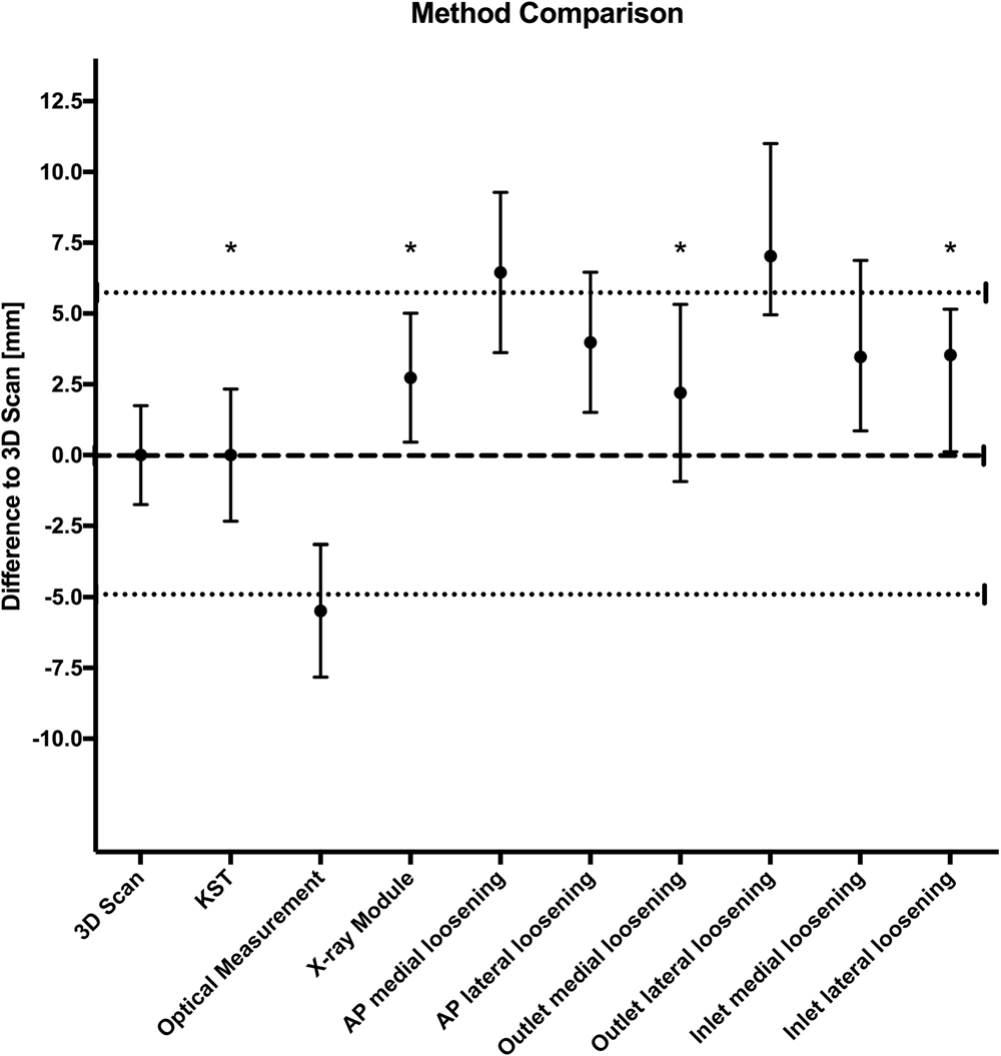
Method comparison. A mean value of the three repetitions of each observer for each pelvis and measurement method was calculated. These generated mean values of each measurement method were subtracted from the mean value of the 3D scan for each pelvis and observer. Subsequently, these difference values were averaged and presented as mean and 95% CI. Extreme values of the 3D scan are indicated by the dotted lines. KST K wire measured screw turn back. *Methods considered equal to the 3D scan.

To estimate methodological identity, we used the extreme values of the 3D scan (−4.9 mm; 5.7 mm) to construct its “equivalence interval”. Comparing the absolute differences of the measurement methods yielded values presented in Supplementary Table S4 and Fig. 4. This analysis revealed the K-wire measured screw turn back equivalent to the 3D scan and supports the use of the 3D scan as suitable “gold standard” (Fig. 4). Furthermore, the X-ray module, outlet medial loosening and inlet lateral loosening were identified as equivalent to the 3D scan. The other methods were out of the predefined range.

Based on the 95% CI of each method, the minimal measurable screw extrusion was calculated as followed: 3D scan 1.8 mm, optical measurement and X-ray module 2.3 mm, AP medial loosening 2.8 mm, AP lateral loosening 2.5 mm, outlet medial loosening 3.1 mm, outlet lateral loosening and inlet medial loosening 3 mm, inlet lateral loosening 2.5 mm.

## Discussion

Compared to 3D scan, the most equivalent estimation of SI screw turn back was assessed by the application of the X-ray module and in fluoroscopy by measuring the inlet lateral loosening and the outlet medial loosening. These techniques yielded a deviation less than 5 mm compared to the 3D scan.

Percutaneous SI screw fixation is a common procedure for the fixation of unstable fractures of the pelvic ring^24–28^. A loosening rate of 2–20% for SI screws was reported^1–3,7,8^. Especially in the elderly population the loosening rate might rise up to 20%^8^. Radiography in AP, inlet and outlet projection are used to assess the instability of pelvic ring fractures^29–31^. Postoperatively, these radiographs are used to grade fracture reduction of the pelvic ring^32,33^. After mobilization, radiographs might reveal SI screw loosening indicating a possible persistent instability or non- union^1–3,8^. However, radiographic SI screw loosening was not always symptomatic, especially if there is no progression of the loosening^1,8^. Radiolucent lines, cup migration or gaps indicating cup loosening following hip arthroplasty^34,35^ or other criteria for implant loosening were previously not established on the pelvic ring possibly due to the impeded visualization of the posterior pelvic ring^36,37^.

Currently, there are no quantitative data on the assessment of SI screw loosening available. Recent studies generally stated if the SI screw is loosened or not^1–3,8^, therefore the definition of the critical SI screw loosening is not possible. To our best knowledge, no quantitative measurement techniques are published yet. Probably, the missing quantitative data are related to the impaired visualization of the posterior pelvic ring^36,37^ and the non- routinely performed CT scans.

Radiographs were found to be less accurate compared to CT determining the anteversion of the cup following hip arthroplasty^38^. Though the registration of a 3D model to plain radiographs and the subsequent determination of the acetabular cup position yielded comparable results, the CT scan remains the “gold standard” for the determination of acetabular cup position^39^. The CT was also superior in the assessment of implant loosening following ankle replacement defined as osteolytic areas, when compared to radiographs^40^.

Due to individual sacral and lumbar anatomy, the angulation for optimal visualization of pelvic anatomy in AP, inlet and outlet radiographs is variable^41–45^. This leads to a different projection of the posterior pelvic ring and individualized outlet and inlet projection angles were proposed^46,47^. However, these individualized angles are difficult to obtain in daily routine. In the present study, the very good ICC (1) and ICC (A,1) for the inlet lateral loosening were not affected by the missing adaption of angulation for the inlet fluoroscopic view. In contrast, the missing adaption of the angulation might explain the worse ICC (A,1) of the outlet medial loosening though it was less than 5 mm different from 3D scan and yielded equivalence to the 3D scan. Thus, the impaired visualization of the bony structures used for the measurements might led to the differing values between the observers^42,48^. The good results of the inlet lateral loosening might be resulted from the consistent upper SI joint projection in the inlet view irrespective to the pelvic tilt. Nonetheless, all these parameters are sensitive to patient positioning, i.e. when the patient lies oblique on the table (symphysis is not centred on the sacral midline), the distances might be distorted.

Radiographic evaluation of the pelvic ring in the elderly is impaired and CT is recommended for fracture detection^36,37^. Recently it was shown that the fluoroscopy after SI screw fixation is insufficient to exclude malpositioning and a postoperative CT scan is required^49^.

In consideration of these data, also a possible impairment of SI screw loosening detection in radiographs might be discussed. Hence, in the present study radiographic measurements were compared to multi-dimensional fluoroscopy from a 3D image intensifier as “gold standard”. Indeed, 3D image intensifier based 3D scans are inferior compared to CT, but they still reveal satisfactory results regarding osteosynthesis position as reported for malpositioning rates of SI screws^50–52^. The improvement of multi-dimensional fluoroscopy by 3D image intensifiers allows appropriate evaluation of implant position and fracture reduction comparable to a CT scan^17,53^. We therefore considered the 3D scan as “gold standard” in the present study.

Beside an accurate determination of the implant loosening, a good radiographic measurement method should also display appropriate ICC (1) and ICC (A,1)^10^. Intrarater reliability (ICC (1)) was for two observers very good using the majority of measurement techniques. Possibly, the data of the remaining observer led to the worse ICC (A,1) determined especially for the 3D scan and X-ray module. In addition, the worse ICC (A,1) for those methods might be caused by the higher individual observer’s choice reducing the reliability^11^. Here, the observer decides independently the slice for measurement in the 3D scan and for optimal sizing as well as accuracy of the contour-based registration in the X-ray module possibly affecting the ICC (A,1).

All fluoroscopic measurements irrespective to their projection (AP, outlet, inlet) for the lateral loosening displayed a very good ICC (A,1), but only the X-ray module, outlet medial loosening and inlet lateral loosening yielded equivalence to the 3D scan. The AP view displayed at least good ICC (1) and ICC (A,1). However, no equivalence to the 3D scan and a higher absolute difference to 3D scan compared to other fluoroscopic measurements were detected. This might be caused by the disguised anatomical landmarks in the AP view, as previously discussed^54^.

Beside the opportunity reducing the choices of the observer, computer-based registrations and their improvement may increase the reliability^55^.

Our results are limited by the small sample size, especially for the fluoroscopic measurements (n = 5) leading to increased deviations compared to the remaining methods. The fluoroscopic measurement techniques presented better results due to the removal of internal organs and missing gas known to impair the visualization of the posterior pelvic ring^36,37^. Thus, future studies are needed to support the presented findings in a clinical setup and prove their validity. The measurements of fluoroscopies following 3D model registration might also increase the reliability due to the correct scaling which might be absent in the clinical practice except by the introduction of a scaling reference^56^. In the present study, the reference was digitally implicated by the registration to the 3D model and the results should not be impaired. But for clinical studies a reference should be included in radiographs. Furthermore, the clinically relevant SI screw extrusion remains unclear. The optical measurement may be underestimated related to the “false” higher values measured digitally; however using the here used approach we were also able to investigate the reliability of this method and in future studies we also recommend to analyse the validity and reliability of calliper based measurements. Moreover, besides SI screw turning back, also a cutout of SI screws or a washer penetration occurs^16^. This implant failure mode should also be evaluated; however the SI screw turn back is the most common observed failure and the only one which might be clinically inconspicuous or indicate persistent instability underlining the need for a quantitative differentiation^1,3^.

Although the X-ray module displayed the smallest deviation to the 3D scan, at least good ICC (1) and an equivalence to the 3D scan, the ICC (A,1) was poor. In contrast, determining the SI screw loosening as inlet lateral loosening showed beside a small deviation to the 3D scan, very good ICC (1), equivalence to the 3D scan also a very good ICC (A,1). Thus, using this method SI screw loosening can be monitored temporally and reduces the radiation for the patient by decreasing the need for CT scans. Moreover, the here presented results indicate the need for outlet and inlet views in the clinical follow- up after SI screw fixation. Furthermore, the X-ray module might be utilized to plan and control SI screw placement as well as to grade fracture reduction in radiographs after introducing reliable anatomical landmarks as known from hip arthroplasty for a faster registration^39,49^. In future studies the here presented measurement techniques should be validated in large clinical trials to reveal the clinically relevant SI screw loosening and correlate SI screw loosening to the functional outcome. Thus, the clinical consequence of SI screw loosening might be revealed. Here, we recommend measuring the lateral loosening in inlet radiographs.

## Supplementary information

Supplementary Table S1-4_Clean

## Data Availability

The datasets generated and analysed during the current study are available from the corresponding author on reasonable request.
