# Supplementary material for: Intra- and interrater reliabilities and a method comparison of 2D and 3D techniques in cadavers to determine sacroiliac screw loosening
Source: Sci Rep. 2019 Feb 28;9:3141. doi: 10.1038/s41598-019-40052-4 (PMC6395688; doi:10.1038/s41598-019-40052-4)
Supplement: Supplementary file 1 — Supplementary Table S1-4_Clean [file 41598_2019_40052_MOESM1_ESM.docx]

**Intra- and interrater reliabilities and a method comparison of 2D and 3D techniques in cadavers to determine sacroiliac screw loosening**

Philipp Pieroh, Maximilian Lenk, Tim Hohmann, Ronny Grunert, Daniel Wagner, Christoph Josten, Andreas Höch, Jörg Böhme

Supplementary Table S1: Baseline data of pelvic specimen. Mean values ± standard deviation for the age and k – wire measured screw turn back as well as the gender- (female [F] and male [M]) and side (left [L] and right [R]) of SI screw loosening distributions are presented in the captions for all tested tissue samples (Σ).

| **Specimen** | **Age [years]** | **Gender** | **Cause of Death** | **Side of unlocked screw** | **K- wire measured turn back [mm]** |
| --- | --- | --- | --- | --- | --- |
| **Σ = 9** | 85.89 ± 4.96 | F:M (3:6) |  | L:R (4:5) | 18.9 ± 3 |
| 1 | 76 | M | Septic Shock | L | 16.0 |
| 2 | 90 | F | Chronic Cardiac Failure | R | 19.0 |
| 3 | 84 | M | Non Traumatic Intracranial Bleeding | R | 22.0 |
| 4 | 91 | M | Unclear | L | 19.0 |
| 5 | 89 | F | Chronic Cardiac Failure | L | 22.5 |
| 6 | 90 | M | Cardiac Arrest | R | 16.0 |
| 7 | 87 | M | Chronic Cardiac Failure | R | 22.0 |
| 8 | 81 | M | Lung Cancer | R | 14.0 |
| 9 | 85 | F | Chronic Cardiac Failure | L | 19.5 |

Supplementary Table S2: SI screw turn back determined using the 3D scan, optical measurement and X- ray module, n=9. Mean ± SD and ranges are indicated in millimeters [mm].

| **Specimen** | **3D Scan [mm]** | | **Optical Measurement [mm]** | | **X-ray module [mm]** | |
| --- | --- | --- | --- | --- | --- | --- |
|  | *Mean ± SD* | *Range* | *Mean ± SD* | *Range* | *Mean ± SD* | *Range* |
| 1 | 15.8 ± 0.8 | (14.6-16.8) | 23.1 ± 0.6 | (22.1-24.4) | 14.3 ± 2.1 | (10.7-17.1) |
| 2 | 17.6 ± 0.8 | (16.5-18.6) | 26.7 ± 1.3 | (24.1-28.1) | 20.4 ± 1.6 | (17.0-23.1) |
| 3 | 19.9 ± 1.0 | (17.9-21.1) | 30.4 ± 1.3 | (28.4-32.3) | 15.3 ± 2.4 | (12.0-18.7) |
| 4 | 18.7 ± 0.2 | (18.3-19.0) | 23.4 ± 1.7 | (21.2-26.1) | 16.1 ± 3.5 | (10.9-19.8) |
| 5 | 21.7 ± 3.4 | (19.4-30.8) | 24.7 ± 1.5 | (21.8-26.9) | 16.7 ± 4.8 | (11.3-22.1) |
| 6 | 19.3 ± 4.1 | (13.6-22.9) | 28.4 ± 4.5 | (20.6-33.8) | 13.0 ± 1.8 | (11.0-16.2) |
| 7 | 23.9 ± 0.5 | (23.2-24.7) | 30.0 ± 2.9 | (24.2-34.2) | 21.2 ± 2.2 | (18.0-24.8) |
| 8 | 18.1 ± 3.4 | (13.6-21.4) | 14.8 ± 1.2 | (12.8-16.8) | 14.0 ± 3.7 | (8.8-20.6) |
| 9 | 20.7 ± 0.9 | (19.7-21.8) | 22.4 ± 2.2 | (18.9-25.3) | 20.4 ± 8.3 | (12.6-34.1) |

Supplementary Table S3: SI screw turn back measured by fluoroscopy, indicated in millimeters [mm].

|  | **AP** | | | | **Outlet** | | | | **Inlet** | | | |
| --- | --- | --- | --- | --- | --- | --- | --- | --- | --- | --- | --- | --- |
|  | *medial* | | *lateral* | | medial | | *lateral* | | *medial* | | *lateral* | |
| Specimen | Mean ± SD | Range | Mean ± SD | Range | Mean ± SD | Range | Mean ± SD | Range | Mean ± SD | Range | Mean ± SD | Range |
| 5 | 14.1 ± 3.6 | (5.0-18.1) | 16.2 ± 2.2 | (11.4-19.0) | 20.2 ± 4.1 | (13.2-23.4) | 8.3 ± 2.3 | (3.8-10.7) | 14.8 ± 8.0 | (1.8-23.5) | 16.9 ± 2.2 | (11.6-18.8) |
| 6 | 13.2 ± 1.6 | (10.4-15.8) | 10.2 ± 1.2 | (8.8-12.0) | 12.6 ± 1.1 | (10.3-13.7) | 11.6 ± 1.2 | (9.6-13.8) | 23.7 ± 7.1 | (13.1-34.9) | 13.4 ± 2.0 | (9.8-16.3) |
| 7 | 21.6 ± 0.9 | (20.0-23.0) | 20.6 ± 2.1 | (18.0-24.0) | 18.6 ± 2.4 | (15.5-23.2) | 23.6 ± 0.5 | (23.0-24.4) | 16.1 ± 2.9 | (11.1-19.1) | 25.9 ± 1.6 | (22.7-28.0) |
| 8 | 10.8 ± 3.8 | (6.1-16.0) | 9.8 ± 1.4 | (8.2-12.0) | 10.9 ± 1.7 | (8.1-13.6) | 7.7 ± 0.8 | (6.7-9.1) | 13.6 ± 5.7 | (5.2-22.2) | 11.6 ± 1.9 | (8.2-15.1) |
| 9 | 6.2 ± 5.7 | (1.6-17.3) | 21.0 ± 0.8 | (19.8-22.7) | 24.3 ± 9.8 | (13.0-38.4) | 6.5 ± 2.2 | (3.3-9.7) | 10.1 ± 4.3 | (4.9-18.1) | 16.7 ± 2.0 | (14.3-19.5) |

Supplementary Table S4: Values for the Method comparison used in Figure 4.

|  | **Mean [mm]** | **95%CI [mm]** | | **Extreme values [mm]** | |
| --- | --- | --- | --- | --- | --- |
| **Method** |  | *lower* | *upper* | *Minimum* | *Maximum* |
| 3D Scan | 0 | -1.8 | 1.8 | -4.9 | 5.7 |
| K-wire measured Screw Turn back | -0.6 | -3.0 | 1.7 |  |  |
| Optical Measurement | -5.5 | -7.8 | -3.2 |  |  |
| X-ray Module | 2.7 | 0.5 | 5.0 |  |  |
| *Fluoroscopic measurements, each n=5* | | | |  |  |
| AP medial loosening | 6.4 | 3.6 | 9.3 |  |  |
| AP lateral loosening | 4.0 | 1.5 | 6.5 |  |  |
| Outlet medial loosening | 2.2 | -0.9 | 5.3 |  |  |
| Outlet lateral loosening | 7.0 | 5.0 | 11.0 |  |  |
| Inlet medial loosening | 3.5 | 0.9 | 6.9 |  |  |
| Inlet lateral loosening | 3.5 | 0.1 | 5.2 |  |  |
